# Maryland and District of Columbia Dental Association

**Published:** 1880-11

**Authors:** 


### EDITORIAL, ETC.

*Maryland and District of Columbia Dental Association.—Annual Meeting.*—The annual meeting of the Maryland and District of Columbia Dental Association was held in the parlors of the Imperial Hotel, Washington, D. C. on the 27th, 28th and 29th of October last. The attendance, while not very large, was made up of intelligent and active workers, and much interest was manifested in the proceedings transpiring from day to day. Most of the sections made reports, which gave rise to considerable and often animated discussion. This was especially the case when the subject of the careless administration of anæsthetics came up for criticism.

Among those visiting the annual meeting from other States, were Drs. Wm. H. Atkinson of N. Y., and Drs. Marshall H. Webb, and Baker and Underwood, of Pennsylvania. Clinics were held by the three last-named gentlemen, with the view of exhibiting the excellence of the electro-magnetic mallet, which certainly, in their hands, did most beautiful work. Dr. Webb used also a burring engine driven by an electric current.

Among others visiting the meeting was Dr. Reese, of New York, who demonstrated the working of his new metal for artificial dentures, the process being a modification of the ordinary stanno-plastic method.

In all respects the meeting was a pleasant and harmonious one, and useful also. The next meeting will, according to the rules, be held in Baltimore, and the time of meeting has been changed to September, as being more convenient, and encroaching less on the busy season. It is understood that the gentlemen who so kindly gave the Association their presence, will visit the Baltimore College of Dental Surgery, and give clinics in the presence of the large class in that school, sometime during the Winter, and that other distinguished members of the profession will be present also, and aid in demonstrating various methods of operating. H.

